# The Burden of Influenza: a Complex Problem

**DOI:** 10.1007/s40471-018-0136-1

**Published:** 2018-02-05

**Authors:** Aubree Gordon, Arthur Reingold

**Affiliations:** 1 Department of Epidemiology, School of Public Health, University of Michigan, Ann Arbor, MI USA; 2 Division of Epidemiology, School of Public Health, University of California, 101 Haviland Hall, Berkeley, CA 94720 USA

**Keywords:** Influenza, Burden, Study design, Bias,, Etiology

Influenza has been long-recognized as an important cause of morbidity and mortality in human populations, leading to development and use of vaccines intended to reduce consequent health and economic impacts. Since 1918, four influenza A pandemics have caused substantial numbers of illnesses and deaths, but annual epidemics caused by influenza A and B viruses have arguably taken a greater toll. Nevertheless, measuring the influenza virus-related burden of disease poses challenges. While influenza A and B viruses cause acute febrile illness, much of the resulting morbidity and mortality is due not to primary influenza illness but to complications and secondary infections not unique to influenza, although most patients no longer have detectable influenza virus when complications develop. Thus, while influenza may lead to exacerbations of many conditions (e.g., chronic obstructive pulmonary disease (COPD), congestive heart failure, and asthma); increased risk of myocardial infarction, cerebrovascular accident, and death due to diverse causes; and increased fetal loss in pregnant women, determining what proportion of such outcomes are attributable to influenza virus is complex, particularly in infants and frail older adults [1–7]. While influenza virus may increase the likelihood of pneumonia and bacteremia caused by *S. pneumoniae*, *H. influenzae b*, and *S. aureus* (including methicillin-resistant *S. aureus*), these bacterial infections also occur in the absence of influenza virus infection [8–12]. Other challenges to studying the burden of influenza-associated illness and death include lack of access to sensitive and specific diagnostic tests, difficulty obtaining specimens for testing, the unpredictability of influenza epidemics (and pandemics), and the cost and complexity of assembling and following large cohorts.

Determining the burden of morbidity and mortality (and resultant economic impacts) attributable to influenza viruses is critical to informing decisions regarding control programs. Particularly in poor countries, where immunization programs are regularly urged to improve coverage of current vaccines while adding new vaccines, decisions concerning the use of influenza vaccines in the absence of a pandemic would benefit from accurate information on the burden of influenza-associated illness and death. Because available influenza vaccines must be formulated and administered annually, their addition to immunization programs in low and even middle-income countries is all the more burdensome, and their value for preventing morbidity, mortality, and economic loss is critical to establish. Here, we review the challenges to estimating the burden of influenza-associated morbidity and mortality. The focus of this review is on illness and death caused by influenza viruses readily transmissible between humans, despite the fact that transmission of avian influenza viruses (e.g., H5N1, H7N9, etc.) to humans can occur and produce life-threatening illness.

## Types of Outcomes

Overwhelming evidence indicates that influenza viruses cause acute infection in the lower and upper respiratory tracts. Often, such illnesses are subdivided for surveillance and disease burden estimation into influenza-like illnesses (ILIs) and severe acute respiratory infections (SARIs). Much of the effort to assess the burden of illness attributable to influenza has relied on surveillance and testing of specimens from patients with ILI, pneumonia, or SARI or from individuals dying of an acute respiratory infection. Other types of studies (e.g., ecologic, modeling) often use “pneumonia and influenza” cases or deaths or all-cause mortality as outcomes.

When individuals are followed over time in either a clinical trial or a cohort study, it is possible to calculate the incidence of acute respiratory illness (ARI), ILI, pneumonia, SARI, and SARI-associated deaths, although large numbers of individuals must be followed to produce reliable estimates of the incidence of SARI, and even larger numbers to produce reliable estimates of SARI-specific mortality. Testing individuals for influenza virus can help determine the incidence of influenza-associated illness and mortality, although attributing an illness or death to influenza virus based on test results may be challenging. Without denominators and ascertainment of all episodes of illness, ILI and SARI surveillance programs can produce information regarding the proportions of such medically attended illnesses in which there is evidence of influenza virus infection but cannot produce accurate incidence rates. Additionally, such surveillance systems do not detect presentations of influenza virus infection that do not meet the surveillance definition (e.g., afebrile).

Furthermore, respiratory infections may account for only a small proportion of the morbidity and mortality caused by influenza. Acute respiratory infections, including those caused by influenza viruses, can exacerbate asthma and COPD, yet in few such episodes is testing for influenza undertaken [2, 3, 5]. Acute influenza can also lead to decompensation of patients with congestive heart failure or diabetes mellitus and to an increased risk of myocardial infarction and cerebrovascular accident, but such patients are rarely tested for influenza [1, 4, 6, 7]. Similarly, influenza virus can cause acute otitis media and is associated with increased risk of Guillain-Barŕe Syndrome [13, 14]. Collectively, these non-specific adverse health outcomes may produce as high a disease burden as ILI and SARI. In addition, delayed effects of infections will be overlooked by standard approaches to assessing influenza-related burden of illness.

## Factors Influencing the Epidemiologic Features of Influenza-Related Illness

Because the burden of illness attributable to influenza in a population depends on who is acquiring it, it is important to consider variations in the descriptive epidemiologic features of influenza. Excluding pandemic influenza, for which the descriptive epidemiologic features of both morbidity and mortality patterns differ, young children experience the highest rates of influenza virus infection and illness and play a central role in transmission of the virus, while it is older adults and young infants who experience a disproportionate risk of severe illness, hospitalization, and death. The presence of underlying chronic conditions (e.g., cardiovascular disease, neuromuscular disease, immunosuppressive medications, and immunodeficiency) increases the risk of severe influenza, so the prevalence of these conditions can influence the proportion of influenza illnesses that are severe or fatal [15, 16]. Conditions such as malnutrition, pregnancy, immediate post-partum period, and obesity (especially morbid obesity) may also increase the risk of severe or fatal influenza [15, 16].

Other factors include climate, which plays an important role in the spread of influenza viruses, with well-documented seasonal peaks of influenza illness and death in populations in temperate climates. The epidemiology in tropical climates is more complex, with diverse seasonal patterns having been described. Demographic factors, such as age distribution, household size, child-spacing, and crowding, likely influence the descriptive epidemiologic features of influenza.

Also important is which influenza virus type (A vs. B) and subtype (H1N1 vs H3H2) or lineage (Yamagata vs. Victoria) is circulating/predominates in a given year and the distribution in the population of immunity to those strains, as a result of prior infection or recent vaccination with an antigenically similar strain. Other factors likely to influence risk of a severe or fatal outcome are the availability of medical care, (e.g., oxygen, ventilatory support), the prompt use of anti-viral drugs, and the use of antimicrobials to treat concurrent or resultant bacterial infections. Also relevant is the use and coverage with vaccines to prevent bacterial infections, (e.g., *S. pneumoniae* polysaccharide and conjugate vaccines, and *Haemophilis influenza* type b (Hib) vaccine), which not only prevents secondary bacterial infections in individuals with influenza but appears also to prevent a proportion of viral pneumonias [17]. Thus, many constantly evolving factors influence the descriptive epidemiological features of influenza-related morbidity and mortality.

## Diagnostic Tests

While some approaches to estimating the burden of influenza-associated illness and mortality do not rely on the use of diagnostic tests, studies of ILI, SARI, and pneumonia almost invariably collect and test specimens. Many older testing methods lacked sensitivity and/or specificity or were difficult to perform. Use of acute and convalescent sera to detect rising antibody titers has always been limited by the difficulty of obtaining these samples, particularly in infants and children. The widespread use of RT-PCR-based and rapid, point-of-care diagnostic tests has revolutionized the study of the role of influenza virus in respiratory infections. While rapid, point-of-care diagnostic tests certainly have their uses, it is important to note that they are generally less sensitive then molecular diagnostic methods [18, 19]. Although RT-PCR has become the standard method used in such studies, studies that have also incorporated serologic testing of patients hospitalized with pneumonia have shown that substantial proportions of influenza-associated cases were detected only by serologic testing [20, 21].

## Detection of Multiple Pathogens

The use of multiplex panels to test respiratory samples for multiple pathogens has illuminated the difficulty of assessing the etiology of respiratory infections. When multiple pathogens are detected, it is unclear if one pathogen is causing the illness or if collectively they are responsible for the illness. Viruses detected may be the immediate cause (e.g., primary viral pneumonia), on the causal pathway or non-causal (e.g., prolonged viral shedding or incidental infection). While many respiratory viruses, including influenza, have been found in asymptomatic individuals [22, 23], the prevalence of influenza virus is typically higher in cases than controls [22]. However, analyzing a single specimen may still not identify an agent involved either earlier or later in the disease process. Studies that test for a single virus or fail to include community controls are likely to misattribute (and possibly overestimate) the number or incidence of illnesses caused by that virus.

## Causal Inference

Since the dissemination of Koch’s Postulates, we have developed a more nuanced understanding of the roles of host and environmental factors that, together with the characteristics of the microbial agents, combine to either produce disease or not. Attempts to develop an alternative set of criteria upon which to base the attribution of a case to a microbial agent have not produced a universally accepted substitute. As we come to understand what a small proportion of the human microbial flora can be cultivated and the effects of the microbiome on health, the need for better criteria for ascribing a causal role in disease to one or more microorganisms has increased.

Many microorganisms, including diverse viruses and bacteria, can be present in the respiratory tract without any discernible effects on health. Regardless of the duration of such infections, that such microorganisms can be present in the respiratory tracts of asymptomatic individuals complicates the causal interpretation of detecting these agents in respiratory specimens from symptomatic individuals. Even specimens from the lower respiratory tract can contain microorganisms that are not harmful. One approach to this problem is to test specimens from non-ill individuals (i.e., controls) enrolled contemporaneously, which can provide useful information on the prevalence of asymptomatic respiratory infection with various microorganisms. The selection of controls, however, has to be carefully planned and executed to avoid selection biases.

## Case Definitions

The findings concerning which microorganisms are found in respiratory samples vary with the type, anatomical site, and severity of infection. Consequently, if results from diverse studies, locations, and time periods are to be usefully considered, standardized case definitions must be used. Recently, the World Health Organization (WHO), Centers for Disease Control and Prevention (CDC), and researchers have developed case definitions for studies of ILI and SARI. In 2015, WHO set out the following case definitions [24] for use in sentinel surveillance programs:

## Influenza-Like Illness

An acute respiratory infection with fever ≥ 38 °C and cough with onset within the last 10 days.

## Severe Acute Respiratory Infections

An acute respiratory infection with history of fever or measured fever ≥ 38 °C and cough with onset within the last 10 days and requires hospitalization.

Many investigators now use these definitions, although frequently modified. Different case definitions are used for young children (< 5 years) and for older children and adults.

Close attention to case definitions is necessary in studies of respiratory infections because the sensitivity, specificity, predictive value positive, and predictive value negative of the case definition(s) may vary. Indeed, the SARI case definition may have varying sensitivity and specificity by age, resulting in the failure to detect many severe acute respiratory illnesses in which there is evidence of influenza virus [25].

## Biases

Given the many variables that influence the risk of influenza, the severity of illness that results, and the likelihood of a fatal outcome, confounding is a potentially serious problem in studies reporting on risk factors for influenza. Important possible confounders in such studies are age, underlying disease status, and variables related to socio-economic status, including household crowding, education level, and income.

More important in studies attempting to measure the burden of disease attributable to influenza are selection and information biases. Facility-based studies that assess which etiologic agents are present in ill individuals rely on individuals to seek care at facilities where surveillance is in place; such individuals are only a subset of people in the community with the same illness, and many factors influence care-seeking behavior. Thus, those coming to sites with ILI and SARI surveillance may not be representative of those in the community with the same clinical manifestations. This is likely to be less of a problem for severe clinical illnesses (e.g., SARI) than for milder illnesses (e.g., ILI), but the availability of alternative healthcare facilities and the extent to which traditional healers are utilized may influence the amount of selection bias that results. If a specimen requires uncomfortable or invasive collection methods, those agreeing to specimen collection may differ from those who refuse, possibly introducing additional selection bias, as might other factors that influence whether eligible study subjects agree to provide specimens or information. In addition, in the hospital setting, specimens may not be obtained from the sickest patients, who often are not enrolled in studies or selected for surveillance.

Potentially the most problematic information bias in studies of the burden of illness attributable to influenza is misclassification of individuals regarding the etiologic role of influenza virus in the outcome. Depending on the timing and types(s) of specimens obtained and the laboratory methods applied, studies may underestimate or overestimate the number, proportion, and rate of respiratory illnesses caused by influenza viruses. While RT-PCR testing of respiratory specimens is now the “gold standard” for confirming the presence of influenza virus, the test results may still lead to misclassification of the etiology of illness, depending on when the specimen is obtained during the illness. Serologic testing of acute and convalescent samples is necessary to identify additional infections. Conversely, without information on the prevalence of influenza virus infection among appropriately selected, contemporaneous controls, illnesses might be attributed to influenza virus when it is an “innocent bystander” and not the cause of the illness.

Other biases can occur in ecologic studies of the burden of illness attributable to influenza. Such studies examine trends in mortality from or hospitalizations for selected health outcomes, irrespective of whether cases are tested for influenza. These studies attempt to estimate the numbers or rates of excess illnesses, hospitalizations, or deaths when influenza viruses are circulating, in categories such as “pneumonia and influenza” and “underlying respiratory and circulatory.” Calculations can be made for a given country or region, age group, or year/influenza season, and for when a given influenza virus type or strain predominates. Depending on the health outcomes, these calculations yield either a lower or an upper bound on the impact of influenza-related illness or death. These studies use statistical approaches to control for the effects of other factors that might influence rates of illness, hospitalization, or death (e.g., circulation of other infectious agents) but remain susceptible to the biases that can affect any ecologic study.

### Study Designs

Some key considerations should be taken into account when interpreting the findings from studies examining the burden of influenza-associated respiratory illness.

## Surveillance/Case Series

The most common approach to assessing the role of influenza viruses in acute illnesses has been facility-based surveillance in the outpatient or hospital setting. Depending on the outcome of interest (ILI, SARI, or pneumonia), surveillance may occur at a number of sentinel sites or at all clinical sites serving the population. Clinical and demographic data are collected, including outcome, and specimens are obtained from all or a sample of those seen who meet the case definition(s). The proportions of illnesses/syndromes in which there is evidence of influenza virus infection, either as the only etiologic agent or in combination with other agents, are calculated. These same surveillance systems can help monitor circulating influenza strains and serve as sources of cases (and often influenza-negative controls) for studies of influenza vaccine effectiveness or risk factors.

Unlike ILI and SARI, deaths attributable to influenza have rarely been the focus of epidemiologic studies or surveillance systems, especially in low- and middle-income settings. Among the challenges to surveillance and studies in low-income settings are the occurrence of deaths outside of healthcare facilities and the difficulty in obtaining specimens for testing.

Studies and surveillance systems that report only the proportion of cases with laboratory evidence of influenza virus yield limited information on the burden of illness, although they play an important role in annual influenza vaccine strain selection. It is possible to calculate incidence (often age-specific) of medically attended illnesses or deaths with laboratory evidence of influenza when the data generated are population-based, the population size is known, and information is available on care-seeking behaviors by ill individuals in the community. The latter is particularly needed when the outcome is mild (e.g., ILI) or when those with severe illness may not have access to or seek care from western-style providers. Adjustment for potential confounding factors (e.g., travel time to a healthcare provider) can improve the accuracy of such calculations.

As described above, case definition and choice of diagnostic tests may influence the observed incidence of influenza-associated respiratory illnesses and deaths. Also important is consistent data collection across multiple years or influenza seasons, given the substantial variability in circulating types and strains of influenza virus and in coverage and effectiveness of influenza vaccines. Finally, the absence of information on the contemporaneous prevalence of influenza virus infection among comparable non-ill individuals in the community may limit causal conclusions regarding influenza viruses and respiratory illnesses.

## Case Series with Contemporaneous Controls (Case-Control Studies)

Case series may incorrectly attribute an episode of ILI or SARI to a microorganism that, while present in the respiratory tract, is not the cause of the illness. To further investigate the etiologic role of viruses detected in patients with respiratory illness, case-control studies that compare the prevalences of various viruses in cases with the prevalences in controls have been conducted. Studies may report simple prevalences of a given virus, calculate odds or risk ratios, or assert a more causal interpretation by presenting the attributable proportion total or the attributable proportion exposed. Because these studies have generally found very low prevalences of influenza viruses among controls, the odds or risk ratios reported have been high, the “adjusted prevalences” of influenza viruses among cases have been only marginally lower than the unadjusted prevalences, and the attributable fraction has been high (e.g., 80–90%) [26–29].

We urge caution in interpreting the results of such case-control studies. Matching controls to cases closely on age and time is critical. Even a short delay in enrollment may affect the prevalence of respiratory viruses in the community. Also, the criteria for enrolling controls are important. Most studies have limited controls to individuals who were asymptomatic for the 14 days prior to enrollment and sampling. Few studies have followed up controls to assure they remain asymptomatic. It is unclear whether these approaches to identifying controls, often from individuals coming to healthcare facilities, produce controls representative of the source population of the cases.

The optimal criteria for selecting control subjects remain the subject of debate, and many case-control studies have enrolled convenience samples as controls. “Pathogen load,” as determined by quantitative PCR, might be useful to measure and compare between cases and controls, although this approach is likely to be informative only if influenza virus is detected in a non-negligible proportion of controls. In a recent study of the association of cycle threshold (CT) values with upper respiratory infection, outpatients and inpatients both had lower CT values (i.e., a higher viral load) than influenza-positive asymptomatic controls. However, there was no clear relationship with disease severity; outpatients had, on average, lower influenza CT values than did inpatients [30]. Results concerning the relationship between CT value and clinical severity from other studies have been mixed [31–34].

## Cohort Studies

Cohort studies have advantages, including measurement of risk factors before the outcome occurs and the ability to study multiple outcomes, and disadvantages, such as cost, length of time to complete, and inefficiency for rare outcomes. The control group of an experimental study can also be used as a “cohort” to measure incidence of various outcomes. To capture episodes of respiratory illness that might not lead to a visit to a health provider, subjects must have frequent active follow-up, e.g., weekly or bi-weekly household visits. Because of the expense and logistical challenges, such studies have been conducted infrequently, but those with active follow-up have reported higher levels of influenza illness and the highest estimates of the burden of influenza [35].

## Nested Case-Control Studies

Case-control studies conducted within a well-defined, longitudinal cohort, can yield incidence of the outcome(s) under study and allow evaluation of risk factor data from the overall cohort. In a nested case-control study, not only is it possible to calculate incidence rates of ILI and SARI in which influenza virus is detectable, but by making comparisons to appropriately selected controls within the cohort, the proportions of such illnesses attributable to influenza may be calculated. However, appropriately apportioning cases when multiple possible etiologic agents are present remains a challenge.

## Modeling Studies and Time Series Analyses

The previous study designs all involve testing specimens from patients with evidence of a respiratory infection for influenza virus. As discussed earlier, such illnesses comprise only a fraction of the sequelae likely attributable to influenza. However, other sequelae, such as COPD exacerbation and myocardial infarction, can have other etiologies, and testing for influenza virus is rarely undertaken in such cases. Consequently, other techniques can be used to estimate overall influenza-associated morbidity or mortality.

One approach uses statistical models to estimate excess deaths or hospitalizations when influenza virus(es) are circulating, compared to periods with no circulating influenza. Such studies have usually been done for high-income countries but can be performed for any region where the circulation of influenza viruses has a defined seasonality or virological results are available to inform models [36–38]. These models ideally account for the circulation of other seasonal microorganisms that cause respiratory infections (e.g., RSV). Models are more difficult to construct for populations in which influenza viruses circulate throughout the year or if the seasonality is poorly characterized or unpredictable. These models require reasonably reliable population-level vital statistics data, including information on at least broad categories of primary and underlying causes of death, lacking in many countries. Two categories of underlying causes of death are generally examined, “pneumonia and influenza” and “respiratory and circulatory”; the former estimates a lower bound and the latter an upper bound on the number or rate of influenza-associated deaths or hospitalizations. These data, stratified by age, can estimate years of life lost attributable to influenza-associated mortality, including by different influenza virus types or strains [39].

Two examples of modeling studies include the Child Health Epidemiology Reference Group and the Global Burden of Disease (GBD) Study ([40–42]. These efforts focused on estimating burden of influenza-related pneumonia/lower respiratory infections but made no attempt to estimate the burden of illness or death attributable to influenza in other disease outcomes. As with any modeling effort, the model output is dramatically affected by the model and the inputs used, as demonstrated by the substantial difference in the estimated annual numbers of deaths in children < 5 resulting from a change in the modeling approach used to produce GBD estimates between 2010 and 2015(119,000 in GBD2010, but only 10,200 in GBD2015) [40, 42].

Factors that may affect burden estimates from modeling studies include the choice of model, time period covered, level of aggregation of data, quantity and quality of virological data available, and use of overall or strain-specific virological data.

Various other modeling approaches used to estimate the amount of mortality or excess mortality “attributable” to influenza virus do not require testing specimens from patients with the diverse other medical conditions plausibly caused or exacerbated by influenza. When carefully and thoughtfully designed, analyzed, and interpreted, such studies can add to what is known about the health impacts of influenza, at least with regard to mortality and hospitalizations. However, a number of cautions are warranted. The paucity of reliable data from some regions (e.g., Africa and South Asia) limits the ability to make reliable local estimates. Second, because many recent modeling efforts have focused on the 2009 pandemic, the results may not be applicable in the context of annual influenza epidemics, or even to future pandemics [43–45]. Annual influenza epidemics have age-specific morbidity and mortality patterns that are markedly different from those seen in influenza pandemics, and future pandemics are likely to be caused by a different influenza virus, as well as occur in a different setting with regard to the distribution in the population of pre-existing immunity to the influenza virus causing the pandemic. Finally, studies of mortality or excess mortality, while capturing an important component of the burden of influenza-associated illness clearly do not capture morbidity.

## Vaccine Probe Studies

Given a vaccine of known efficacy against illness caused by the specific etiologic agent, a vaccine probe study allows the calculation of the proportion of such illnesses caused by that etiologic agent (i.e., etiologic fraction), as well as the difference in the incidence of the illness between vaccinated and unvaccinated people (i.e., vaccine-preventable disease incidence) [46]. If the efficacy of the vaccine against illness caused by the etiologic agent is not well-defined, a nested study within the vaccine probe study is needed to generate that information. Vaccine probe studies have proven to be very informative with regard to estimation of the proportion of pneumonias attributable to bacterial pathogens (e.g., *Hib* and *S. pneumonia*) and how much illness and death can be prevented with these bacterial conjugate vaccines [47–50]. Vaccine probe studies have been used rarely to investigate influenza viruses [51–54]. Influenza vaccine probe studies are a potentially attractive approach to estimating what proportions of various health outcomes are attributable to influenza and how many illnesses (and possibly deaths) can be prevented by influenza vaccines.

However, given the variation in the effectiveness of influenza vaccines, as well as recent evidence that live-attenuated influenza vaccines have substantially lower efficacy than initially demonstrated, issues related to influenza vaccine probe studies require careful consideration. Such studies will have to be of adequate size to produce useful estimates of the amount of vaccine-preventable illness (and possibly deaths), include nested studies of vaccine efficacy against laboratory-confirmed influenza infections, and be carried out in multiple settings and across multiple influenza seasons. Such studies will be large, complex, and expensive. Given the unpredictability of influenza, even large and expensive studies of this type may fail to provide robust estimates of the burden of illness attributable to influenza, and these estimates may or may not be applicable in other settings, seasons, and patient populations.

## Discussion

With finite resources to devote to health promotion and disease prevention, one reason to establish the magnitude of influenza-associated morbidity and mortality is to quantify the proportion of such outcomes potentially preventable through immunization. In low- and middle-income countries, annual influenza immunization has not been considered a high priority, although the 2009 H1N1 influenza pandemic fostered new interest in this area. Much of the subsequent research and surveillance activity has focused on ILI and SARI, two well-established outcomes of influenza infection. As alluded to above, however, substantial evidence suggests that influenza can produce a diversity of acute adverse health outcomes and longer-term effects. Other than modeling/time series studies and vaccine probe studies, research on the role of influenza virus as an etiologic agent answers a narrow question concerning respiratory illnesses and typically cannot, by design, provide information on its possible role in other illnesses.

The design and the interpretation of the results of studies of the etiologic agents responsible for cases of respiratory illness are complicated, and generalizing their results to larger populations or to other time periods is fraught with challenges related to choice of diagnostic technology, case definition and control selection, and analytic model. Attributing a given illness to influenza virus when multiple microorganisms are detected in a single specimen remains a challenge. To calculate accurate incidence rates of influenza-associated ARI, ILI, pneumonia, or SARI, approaches that take into account care-seeking behaviors and the factors that influence such behaviors, together with accurate information concerning the size and composition of the source population, are needed.

Also important in summarizing what is known about the burden of illness attributable to influenza virus is the variability in that burden. Unlike some infectious diseases, the burden of influenza-associated illness and death is highly variable, by geographic region and over time. There is substantial variation in the overall and the age-specific burden of influenza-related morbidity and mortality, depending on which influenza virus(es) predominate. Not only are severity of illness and the age groups at greatest risk of severe illness and death different in the context of pandemic vs. annual epidemic influenza but they also differ, depending on the type or subtype of influenza that predominates. Thus, the findings concerning the burden of illness vary by year and by season, at least in temperate climates, where influenza shows marked seasonal peaks. Thus, data collected over multiple years and across all seasons need to be collected and compiled for studies of influenza-associated illness to be most useful.

Other factors are likely to influence the amount of influenza-related morbidity and mortality and its distribution within a population. In countries administering influenza vaccines to a sizeable proportion of the population, influenza vaccine coverage and vaccine effectiveness against predominant strains, both of which can vary substantially, will influence the findings of studies of influenza burden of disease. Use of other vaccines, particularly Hib and *S. pneumoniae* polysaccharide and conjugate vaccines, is likely to have an impact on the burden of disease attributable to influenza, to the extent that a preceding or concurrent influenza virus infection predisposes to bacterial pneumonia. The prevalences of diverse chronic conditions, particularly untreated HIV infection, but also such conditions as obesity and malnutrition, are likely to influence the severity of and risk of dying associated with influenza virus infection. And demographic factors are plausible modifiers of the distribution of influenza infection and influenza-related illness in the community. To be most informative, studies must not only cover multiple seasons/years but represent a number of different geographic settings and epidemiological situations.

## Summary

Overall, deriving precise and reliable estimates of the burden of illness attributable to influenza virus infection, either by country/region or globally, is made difficult by numerous methodologic challenges. No single study or study design can provide all of the information needed to estimate influenza-related morbidity and mortality, although well-designed and executed vaccine probe studies of sufficient size can add substantially to our knowledge base.

## References

[1] Corrales-Medina VF, Alvarez KN, Weissfeld LA, Angus DC, Chirinos JA, Chang CC (2015). Association between hospitalization for pneumonia and subsequent risk of cardiovascular disease. JAMA.

[2] Johnston SL, Pattemore PK, Sanderson G, Smith S, Lampe F, Josephs L, Symington P, O'Toole S, Myint SH, Tyrrell DAJ, Holgate ST (1995). Community study of role of viral infections in exacerbations of asthma in 9-11 year old children. BMJ.

[3] Nicholson KG, Kent J, Ireland DC (1993). Respiratory viruses and exacerbations of asthma in adults. BMJ.

[4] Warren-Gash C, Smeeth L, Hayward AC (2009). Influenza as a trigger for acute myocardial infarction or death from cardiovascular disease: a systematic review. Lancet Infect Dis.

[5] Wedzicha JA (2004). Role of viruses in exacerbations of chronic obstructive pulmonary disease. Proc Am Thorac Soc.

[6] Warren-Gash C, Bhaskaran K, Hayward A, Leung GM, Lo SV, Wong CM, Ellis J, Pebody R, Smeeth L, Cowling BJ (2011). Circulating influenza virus, climatic factors, and acute myocardial infarction: a time series study in England and Wales and Hong Kong. J Infect Dis.

[7] Warren-Gash C, Hayward AC, Hemingway H, Denaxas S, Thomas SL, Timmis AD, Whitaker H, Smeeth L (2012). Influenza infection and risk of acute myocardial infarction in England and Wales: a CALIBER self-controlled case series study. J Infect Dis.

[8] McCullers JA (2006). Insights into the interaction between influenza virus and pneumococcus. Clin Microbiol Rev.

[9] Weinberger DM, Simonsen L, Jordan R, Steiner C, Miller M, Viboud C (2012). Impact of the 2009 influenza pandemic on pneumococcal pneumonia hospitalizations in the United States. J Infect Dis.

[10] Shrestha S, Foxman B, Weinberger DM, Steiner C, Viboud C, Rohani P (2013). Identifying the interaction between influenza and pneumococcal pneumonia using incidence data. Sci Transl Med.

[11] Finelli L, Fiore A, Dhara R, Brammer L, Shay DK, Kamimoto L, Fry A, Hageman J, Gorwitz R, Bresee J, Uyeki T (2008). Influenza-associated pediatric mortality in the United States: increase of Staphylococcus aureus coinfection. Pediatrics.

[12] Bhat N, Wright JG, Broder KR, Murray EL, Greenberg ME, Glover MJ, Likos AM, Posey DL, Klimov A, Lindstrom SE, Balish A, Medina MJ, Wallis TR, Guarner J, Paddock CD, Shieh WJ, Zaki SR, Sejvar JJ, Shay DK, Harper SA, Cox NJ, Fukuda K, Uyeki TM, Influenza Special Investigations Team (2005). Influenza-associated deaths among children in the United States, 2003–2004. N Engl J Med.

[13] Vellozzi C, Iqbal S, Broder K (2014). Guillain-Barre syndrome, influenza, and influenza vaccination: the epidemiologic evidence. Clin Infect Dis.

[14] Buchman CA, Doyle WJ, Skoner DP, Post JC, Alper CM, Seroky JT, Anderson K, Preston RA, Hayden FG, Fireman P, Ehrlich GD (1995). Influenza a virus—induced acute otitis media. J Infect Dis.

[15] Mertz D, Kim TH, Johnstone J, Lam PP, Science M, Kuster SP, Fadel SA, Tran D, Fernandez E, Bhatnagar N, Loeb M (2013). Populations at risk for severe or complicated influenza illness: systematic review and meta-analysis. BMJ.

[16] Van Kerkhove MD, Vandemaele KA, Shinde V, Jaramillo-Gutierrez G, Koukounari A, Donnelly CA (2011). Risk factors for severe outcomes following 2009 influenza a (H1N1) infection: a global pooled analysis. PLoS Med.

[17] Madhi SA, Klugman KP (2004). A role for Streptococcus pneumoniae in virus-associated pneumonia. Nat Med.

[18] Merckx J, Wali R, Schiller I, Caya C, Gore GC, Chartrand C, Dendukuri N, Papenburg J (2017). Diagnostic accuracy of novel and traditional rapid tests for influenza infection compared with reverse transcriptase polymerase chain reaction: a systematic review and meta-analysis. Ann Intern Med.

[19] Chartrand C, Leeflang MM, Minion J, Brewer T, Pai M (2012). Accuracy of rapid influenza diagnostic tests: a meta-analysis. Ann Intern Med.

[20] Jain S, Self WH, Wunderink RG, Fakhran S, Balk R, Bramley AM, Reed C, Grijalva CG, Anderson EJ, Courtney DM, Chappell JD, Qi C, Hart EM, Carroll F, Trabue C, Donnelly HK, Williams DJ, Zhu Y, Arnold SR, Ampofo K, Waterer GW, Levine M, Lindstrom S, Winchell JM, Katz JM, Erdman D, Schneider E, Hicks LA, McCullers JA, Pavia AT, Edwards KM, Finelli L, CDC EPIC Study Team (2015). Community-acquired pneumonia requiring hospitalization among U.S. adults. N Engl J Med.

[21] Jain S, Williams DJ, Arnold SR, Ampofo K, Bramley AM, Reed C, Stockmann C, Anderson EJ, Grijalva CG, Self WH, Zhu Y, Patel A, Hymas W, Chappell JD, Kaufman RA, Kan JH, Dansie D, Lenny N, Hillyard DR, Haynes LM, Levine M, Lindstrom S, Winchell JM, Katz JM, Erdman D, Schneider E, Hicks LA, Wunderink RG, Edwards KM, Pavia AT, McCullers JA, Finelli L, CDC EPIC Study Team (2015). Community-acquired pneumonia requiring hospitalization among U.S. children. N Engl J Med.

[22] Rhedin S, Lindstrand A, Hjelmgren A, Ryd-Rinder M, Ohrmalm L, Tolfvenstam T (2015). Respiratory viruses associated with community-acquired pneumonia in children: matched case-control study. Thorax.

[23] Jansen RR, Wieringa J, Koekkoek SM, Visser CE, Pajkrt D, Molenkamp R, de Jong MD, Schinkel J (2011). Frequent detection of respiratory viruses without symptoms: toward defining clinically relevant cutoff values. J Clin Microbiol.

[24] WHO. A manual for estimating disease burden association with seasonal influenza. World Health Organization. 2015.

[25] Makokha C, Mott J, Njuguna HN, Khagayi S, Verani JR, Nyawanda B, Otieno N, Katz MA (2016). Comparison of severe acute respiratory illness (sari) and clinical pneumonia case definitions for the detection of influenza virus infections among hospitalized patients, western Kenya, 2009-2013. Influenza Other Respir Viruses.

[26] Benet T, Sanchez Picot V, Messaoudi M, Chou M, Eap T, Wang J, et al. Microorganisms associated with pneumonia in children <5 years of age in developing and emerging countries: thE GABRIEL pneumonia multicenter, prospective, case-control study. Clin Infect Dis. 2017;12(10). 10.1093/cid/cix378 PMC7108107 28605562

[27] Feikin DR, Njenga MK, Bigogo G, Aura B, Aol G, Audi A, Jagero G, Muluare PO, Gikunju S, Nderitu L, Winchell JM, Schneider E, Erdman DD, Oberste MS, Katz MA, Breiman RF (2013). Viral and bacterial causes of severe acute respiratory illness among children aged less than 5 years in a high malaria prevalence area of western Kenya, 2007-2010. Pediatr Infect Dis J.

[28] Pretorius MA, Tempia S, Walaza S, Cohen AL, Moyes J, Variava E (2016). The role of influenza, RSV and other common respiratory viruses in severe acute respiratory infections and influenza-like illness in a population with a high HIV sero-prevalence, South Africa 2012-2015. J Clin Virol.

[29] Shi T, McLean K, Campbell H, Nair H (2015). Aetiological role of common respiratory viruses in acute lower respiratory infections in children under five years: a systematic review and meta-analysis. J Glob Health.

[30] Fuller JA, Njenga MK, Bigogo G, Aura B, Ope MO, Nderitu L, Wakhule L, Erdman DD, Breiman RF, Feikin DR (2013). Association of the CT values of real-time PCR of viral upper respiratory tract infection with clinical severity. Kenya J Med Virol.

[31] Lee CK, Lee HK, Loh TP, Lai FY, Tambyah PA, Chiu L (2011). Comparison of pandemic (H1N1) 2009 and seasonal influenza viral loads, Singapore. Emerg Infect Dis.

[32] Lee N, Chan PK, Hui DS, Rainer TH, Wong E, Choi KW (2009). Viral loads and duration of viral shedding in adult patients hospitalized with influenza. J Infect Dis.

[33] Li CC, Wang L, Eng HL, You HL, Chang LS, Tang KS, Lin YJ, Kuo HC, Lee IK, Liu JW, Huang EY, Yang KD (2010). Correlation of pandemic (H1N1) 2009 viral load with disease severity and prolonged viral shedding in children. Emerg Infect Dis.

[34] To KK, Hung IF, Li IW, Lee KL, Koo CK, Yan WW (2010). Delayed clearance of viral load and marked cytokine activation in severe cases of pandemic H1N1 2009 influenza virus infection. Clin Infect Dis.

[35] Nair H, Brooks WA, Katz M, Roca A, Berkley JA, Madhi SA, Simmerman JM, Gordon A, Sato M, Howie S, Krishnan A, Ope M, Lindblade KA, Carosone-Link P, Lucero M, Ochieng W, Kamimoto L, Dueger E, Bhat N, Vong S, Theodoratou E, Chittaganpitch M, Chimah O, Balmaseda A, Buchy P, Harris E, Evans V, Katayose M, Gaur B, O'Callaghan-Gordo C, Goswami D, Arvelo W, Venter M, Briese T, Tokarz R, Widdowson MA, Mounts AW, Breiman RF, Feikin DR, Klugman KP, Olsen SJ, Gessner BD, Wright PF, Rudan I, Broor S, Simões EAF, Campbell H (2011). Global burden of respiratory infections due to seasonal influenza in young children: a systematic review and meta-analysis. Lancet.

[36] Serfling RE (1963). Methods for current statistical analysis of excess pneumonia-influenza deaths. Public Health Rep.

[37] Simonsen L, Fukuda K, Schonberger LB, Cox NJ (2000). The impact of influenza epidemics on hospitalizations. J Infect Dis.

[38] Thompson WW, Shay DK, Weintraub E, Brammer L, Cox N, Anderson LJ, Fukuda K (2003). Mortality associated with influenza and respiratory syncytial virus in the United States. JAMA.

[39] Estimates of deaths associated with seasonal influenza—United States, 1976–2007. MMWR Morb Mortal Wkly Rep. 2010;59(33):1057–62. 20798667

[40] Rudan I, O'Brien KL, Nair H, Liu L, Theodoratou E, Qazi S, Lukšić I, Fischer Walker CL, Black RE, Campbell H, Child Health Epidemiology Reference Group (CHERG) (2013). Epidemiology and etiology of childhood pneumonia in 2010: estimates of incidence, severe morbidity, mortality, underlying risk factors and causative pathogens for 192 countries. J Glob Health.

[41] Kovacs SD, Mullholland K, Bosch J, Campbell H, Forouzanfar MH, Khalil I, Lim S, Liu L, Maley SN, Mathers CD, Matheson A, Mokdad AH, O’Brien K, Parashar U, Schaafsma TT, Steele D, Hawes SE, Grove JT (2015). Deconstructing the differences: a comparison of GBD 2010 and CHERG's approach to estimating the mortality burden of diarrhea, pneumonia, and their etiologies. BMC Infect Dis.

[42] Global, regional, and national life expectancy, all-cause mortality, and cause-specific mortality for 249 causes of death, 1980–2015: a systematic analysis for the Global Burden of Disease Study 2015. Lancet. 2016;388(10053):1459–544. 10.1016/S0140-6736(16)31012-1. 10.1016/S0140-6736(16)31012-1 PMC5388903 27733281

[43] Dawood FS, Iuliano AD, Reed C, Meltzer MI, Shay DK, Cheng PY, Bandaranayake D, Breiman RF, Brooks WA, Buchy P, Feikin DR, Fowler KB, Gordon A, Hien NT, Horby P, Huang QS, Katz MA, Krishnan A, Lal R, Montgomery JM, Mølbak K, Pebody R, Presanis AM, Razuri H, Steens A, Tinoco YO, Wallinga J, Yu H, Vong S, Bresee J, Widdowson MA (2012). Estimated global mortality associated with the first 12 months of 2009 pandemic influenza A H1N1 virus circulation: a modelling study. Lancet Infect Dis.

[44] Lemaitre M, Carrat F, Rey G, Miller M, Simonsen L, Viboud C (2012). Mortality burden of the 2009 A/H1N1 influenza pandemic in France: comparison to seasonal influenza and the A/H3N2 pandemic. PLoS One.

[45] Simonsen L, Spreeuwenberg P, Lustig R, Taylor RJ, Fleming DM, Kroneman M, van Kerkhove MD, Mounts AW, Paget WJ, the GLaMOR Collaborating Teams (2013). Global mortality estimates for the 2009 influenza pandemic from the GLaMOR project: a modeling study. PLoS Med.

[46] Feikin DR, Scott JA, Gessner BD (2014). Use of vaccines as probes to define disease burden. Lancet.

[47] Gessner BD, Sutanto A, Linehan M, Djelantik IG, Fletcher T, Gerudug IK (2005). Incidences of vaccine-preventable Haemophilus influenzae type b pneumonia and meningitis in Indonesian children: hamlet-randomised vaccine-probe trial. Lancet.

[48] Klugman KP, Madhi SA, Huebner RE, Kohberger R, Mbelle N, Pierce N (2003). A trial of a 9-valent pneumococcal conjugate vaccine in children with and those without HIV infection. N Engl J Med.

[49] Levine OS, Lagos R, Munoz A, Villaroel J, Alvarez AM, Abrego P (1999). Defining the burden of pneumonia in children preventable by vaccination against Haemophilus influenzae type b. Pediatr Infect Dis J.

[50] Lucero MG, Nohynek H, Williams G, Tallo V, Simoes EA, Lupisan S (2009). Efficacy of an 11-valent pneumococcal conjugate vaccine against radiologically confirmed pneumonia among children less than 2 years of age in the Philippines: a randomized, double-blind, placebo-controlled trial. Pediatr Infect Dis J.

[51] Arnoux S, Weinberger C, Gessner BD (2007). Vaccine-preventable influenza disease burden from clinical trials of Vaxigrip—an inactivated split virion influenza vaccine - supports wider vaccine use. Vaccine.

[52] Phrommintikul A, Wongcharoen W (2012). Examining the potential of the influenza vaccine for secondary prevention: a myocardial infarction vaccine?. Futur Cardiol.

[53] Qureshi H, Gessner BD, Leboulleux D, Hasan H, Alam SE, Moulton LH (2000). The incidence of vaccine preventable influenza-like illness and medication use among Pakistani pilgrims to the Haj in Saudi Arabia. Vaccine.

[54] Zaman K, Roy E, Arifeen SE, Rahman M, Raqib R, Wilson E, Omer SB, Shahid NS, Breiman RF, Steinhoff MC (2008). Effectiveness of maternal influenza immunization in mothers and infants. N Engl J Med.

